# Two sides of the same coin: person-centred systems versus person-centred nursing practice. Theory, barriers and opportunities

**DOI:** 10.1177/17449871241255012

**Published:** 2025-04-16

**Authors:** Amy-Louise Byrne

**Affiliations:** Senior Lecturer and Postgraduate Research Coordinator, School of Nursing, Midwifery and Social Sciences, CQUniversity, Sydney, Australia

**Keywords:** nursing, person-centred care, system redesign

## Abstract

**Background:**

Person-centred care is a well-known concept in nursing practice. It is understood that person-centred care represents a way of providing care, which considers the person’s social, emotional and physical health. Person-centred care is tailored care, which meets individual’s needs, while also including them in decision-making.

**Aims:**

Despite this, the practice of person-centred care can be stifled by system constraints which impact largely on the nursing profession. This paper aims to explore such challenges, highlighting the disconnect between theory and practice.

**Methods:**

Adapted from the work of Fairclough, this discourse analysis critically reviews a cross section of texts related to person-centred care and offers a critique for discussion.

**Results:**

This paper has found a divergence between person-centred *care* (system) and person-centred *practice* (nursing care), highlighting the tension between the theories and practice.

**Conclusion and contribution to nursing:**

This paper highlights not only the challenges but also the opportunities in embedding person-centred care into system design, and considers further changes required to enable nurses to practice care which supports the whole needs of the person. It suggests that leveraging nursing expertise and experience may provide an avenue for system change.

## Introduction

Person-centred care (PCC) is a well-known concept within health. Taking shape through the age of psychiatry, the notion of caring for the whole patient (medical, psychological, psychosocial) made a connection between mind and body working together for wellness (Balint, 1968; Engle, 1977). Initially known as patient-centred care, the term came into focus at the turn of the century, facilitated through the quality and safety movement of healthcare. The Institute of Medicine’s (2001) ‘Crossing the Quality Chasm’ report provided one of the first definitions of the concept, suggesting that PCC included qualities of compassion, empathy and responsiveness to individual needs, values and preferences.

The concept has evolved over time, moving from patient-centred care to person-centred care, and various synonymous concepts in between (e.g. consumer-led, resident-centred, client-centred care). Internationally, the World Health Organization (WHO, n.d.a) describes integrated people-centred care as an approach required to ensure equity in access, quality, responsiveness and participation, efficiency and resilience. In Australia, the concept is considered an

. . .innovative approach to the planning, delivery, and evaluation of health care that is grounded in mutually beneficial partnerships among health care providers, patients, and families. (Australian Commission on Safety and Quality in Healthcare (ACSQHC), 2010: 13)

While the literature is somewhat consistent in its altruistic approach to this concept, and the core makeup of its components (shared decision-making, therapeutic relationship, communication, addressing all elements of the person), there is no one universally adopted definition of PCC (Byrne et al., 2020). This leaves the concept open to interpretation and opens space for tensions between the system within which PCC is located, and the professions that are asked to practise it.

Indeed, despite roots in the medical field, and the system approach described in the previous definitions, PCC is largely positioned within the nursing profession. Gachoud et al. (2012) found a distinct hierarchy of person-centredness among professions, with nurses the most responsible, and medical officers inclined to accept a lower position on delivering such care. In addition, most medical models, particularly general practice and specialist care, are disease-centric, rather than person-centric, due to the business models which dictate billing (Whitty et al., 2020; John et al., 2020). Indeed, modern healthcare is built on the principles of new public management: a neo-liberal ethos that attempts to run public services with private business ideals including economic principles of productivity and efficiency. In addition to this, traditional workforce structures and power are evident, with nurses being positioned as a semi-professional responsible for care and caring, with little power to enact change within an organisation (Ghadirian et al., 2014; Heldal et al., 2019) Traditionally, nurses were viewed as handmaidens, assisting the medical officers with care tasks. While nursing professionalism has expanded in more recent times, the traditional positioning of nursing means that their ability to make decisions and influence wider systemic parameters remains limited (Teresa-Morales et al., 2022). Hence, the practice of PCC can be stifled by system constraints which impact largely on the nursing profession. This paper aims to explore such challenges, highlighting the disconnect between the theory and practice of PCC in Australian nurses.

While some literature uses the terms patient and person interchangeably, this paper chooses to use the term PCC, in recognition that engagement with healthcare providers, as patient, is only one element of a person’s life. In adopting the term person, the paper makes a conscious choice to move away from labels which medicalise and categorise people based on their disease or illness.

## Methods

This paper is a discourse analysis on the topic of PCC in nursing, designed to spark discussion. It takes the position that nurses are part of a healthcare system and looks closely at the barriers which impede PCC and system reform, and the opportunities available. The work of Fairclough (2015) was used, which describes a process of ordering discourse across distinctive social orders to enable investigation and discussion around the divergence and convergence across these. While this is normally achieved by ordering across micro, meso and macro orders, this paper has adapted the framework (Fairclough, 2013, 2015) to compare Australian discourse on PCC into two categories: PCC service requirements and PCC for nurses. The discourses are provided in Table 1 and they were selected based on a cross section of freely available documents related to PCC in Australia.

**Table 1.** Order of discourse.

PCC service requirements	PCC for nurses
• Australian Charter of Healthcare Rights (National) • Australian Commission on Safety and Quality in Healthcare – Person Centred Healthcare Organisations (National) • Clinical Excellence Division – PCC (State)	• International Council of Nurses – Code of Conduct for Nurses (International) • Australian Nursing Standards for Practice (National) • Australian College of Nurses – PCC (National)

Once gathered and reviewed, the discourse was considered against nursing theories to better understand the disconnect between theory and practice (where practice is a product of system influence). Existing literature was integrated to position the argument within the historical, social, cultural and political contexts that impact modern healthcare.

The findings are summarised as PCC (system) versus Person-Centred Practice (PCP), which causes theory dilution at the point of praxis. Importantly, this paper offers opportunities in PCC which can be led by the nursing profession.

### Evolution of person-centred theories and modern healthcare

First, it is important to ground this analysis within the relevant theories pertaining to PCC in nursing. Understanding and nursing the whole person, is certainly not a new concept. Indeed, Abdellah’s patient-centred approaches to nursing were published as early as 1960. Abdellah’s 21 Problems in Nursing Theory made a conscious effort to include the concept of personhood, marrying the medical and social as equal aspects to the nursing care process. The theory was designed around the identification and solution of nursing problems, which centred on knowing one’s patient, teaching the patient and maintaining a therapeutic relationship with patient and family (Abdellah, 1960). Nursing care through this theory is viewed as addressing a series of health needs (e.g. nutrition, elimination, fluid and electrolyte balance), along with psychosocial aspects such as the emotional wellbeing of patients, the expression of feelings, spiritual goals and understanding social problems which influence illness (Abdellah, 1960). This theory caught the attention of nurse academics who questioned how such a theory may be taught to student nurses, and how they could influence the changes required to do so, as one book review from 1960 reads

This new approach [patient-centred care] is not without problems. It seems to me that a period of transition will be needed to enable instructors to comprehend their new role in the new approach . . . This text, the first to spell out with clarity the patient-centred approach to nursing care, is a real contribution to the profession. (Glasoe, 1960: 1439)

What is clear from this, is that nurses were looking at new ways of merging the art and science of nursing, where tasks were a matter of reason and process, and formed part of care which was more socially understood.

Indeed, ‘caring’ began to be viewed as conceptual, and raised questions for nurses such as what does it mean to care? Care was theorised as a process that sat outside of the medically and disease-dominated models and was considered to be holistic, where the person is viewed as a being that exists outside of the medical gaze. This of course occurred in the context of a changing hospital and health system, with Australia moving to the Medicare health system in 1984. This universal system moved healthcare into the realm of federal funding and thus legitimatised the need to fiscally govern healthcare and service provision (National Museum of Australia, 2023). This continues today, with healthcare costs at the top of political agendas (see e.g. the Australian healthcare expenditure reports or strategic frameworks for chronic illness and aged care, considered some of the most costly and vulnerable population groups; Australian Institute of Health and Welfare, 2022a).

Person-centred theories in this space continued to evolve over time, particularly in areas where people receiving care were vulnerable, for example, Kitwood’s (1993) framework for psychological needs of people with dementia, aged care and later theories which applied PCC to people with chronic conditions (see e.g. Hatzichristou et al., 2005).

Around this time, Australian nursing had transitioned to a university-based system, moving away from hospital training. This move was fuelled by several social and political influences including economic reform, the quality and safety movement and the predicted nursing workforce shortage (Grealish, 2012). This form of professionalisation included with it an ideology of life-long learning, where nurses were held to account for their own learning and practice, regardless of the environment in which they worked (Rudge, 2013). Nursing theories such as novice to expert (Benner, 1984) coincided with these changes, perpetuating the idea that nurses can influence quality outcomes where ‘*nursing models [guide] them about what questions they can ask, how they can process the information that is learned, what nursing activity can be included in their care practices’* (Ozdemir, 2019: 1279). At a time when nursing was finding its new place in the university sector, it also needed to adjust practice based on the new public management ethos, providing efficient yet person-centred services.

**Figure 1.**
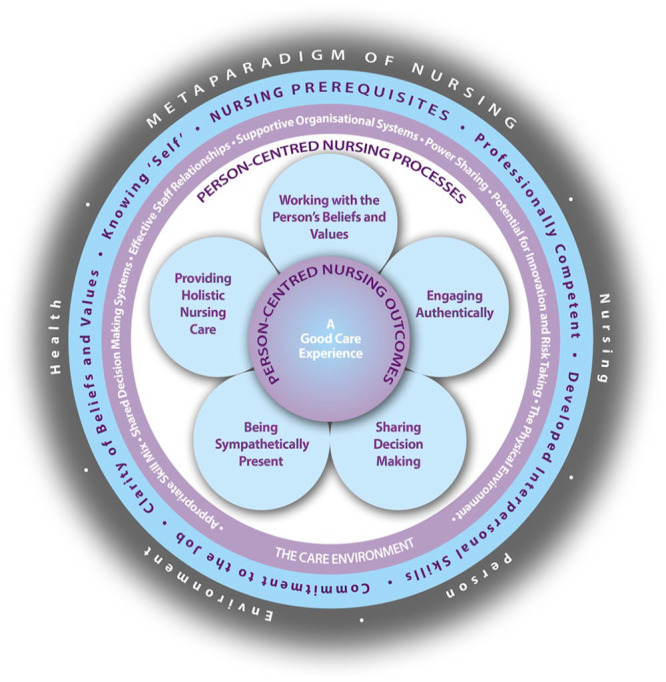
The person-centred nursing framework. Source: (Reproduced with permission from the authors): Dewing, McCormack and McCance (2021).

Modern theoretical iterations of personhood and person-centredness were introduced by McCormack and McCance (2010), who understood this to be a complex mix of a person’s *being.* Theory considered being in relation to other people, being in a social world, being in place (the context of one’s personhood) and being with self (being recognised and respected; McCormack and McCance, 2010). In relation to nursing practice, this enabled the development of one of the most recognisable PCC frameworks, as demonstrated in Figure 1.

These iterations occurred around the same time as the quality and safety movement in healthcare, where health services were required to obtain accreditation, based on several metrics which demonstrate processes and policies which allow for safe patient care. In Australia, the first version of accreditation standards was introduced via the *National Health Reform Act 2011* (2011) Herein, the standards necessitated PCC, defining it as:

The delivery of health care that is responsive to the needs and preferences of patients. Patient-centred care is a dimension of safety and quality. (ACSQHC, 2012)

A closer examination of the second edition of standards will take place below.

PCC and nursing theories evolved together through a complex journey of healthcare and nursing reform. Indeed, through professionalisation, nurses were positioned as a valuable resource to be effectively and efficiently used in the pursuit of PCC, as a health service requirement for accreditation. The examination of discourse continues in the next sections with the two separate concepts: PCC (system) versus PCP.

### Person-centred *care* – the system approach

The emergence and insertion of PCC in health service in Australia, at least discursively, positions it as a system requirement. That is, health services and associated facilities are required to demonstrate a PCC approach to planning and delivery. This positions PCC as a separate concept to that of practice.

The Australian Charter of Healthcare Rights (ACSQHC, 2020) describes the rights of Australian people. The document describes seven fundamental rights: Access, Safety, Respect, Partnership, Information, Privacy and Feedback. An individual has the right to access safe services that meet their needs, while being treated with dignity and respect and being involved in communication and decision-making. From the charter:

[I have the right to] Receive information about services, waiting times and costs . . . [I have the right to] be given assistance, when I need it, to help me to understand and use health information. (ACSQHC, 2020)

The charter legitimises the right to PCC, and elements of cultural safety and access, though it is not specific on how this is achieved. *National Safety and Quality Health Services Standards* (2nd edn., 2021) by the same authors provides broader detail on this. The intention of standard two – Partnering with Consumers, is described as:

To create an organisation in which there are mutually valuable outcomes by having consumers as partners in planning, design, delivery, measurement and evaluation of systems and service, and patients as partners in their own care, to the extent that they choose. (ACSQHC, 2021)

**Figure 2.**
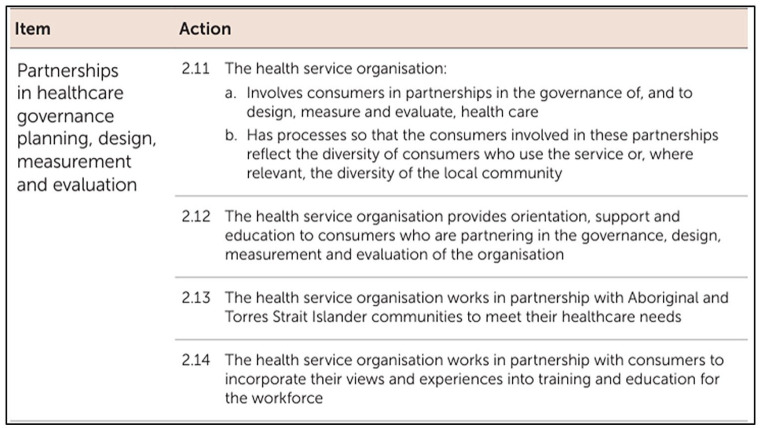
Partnering with consumers in governance, planning, design, measurement and evaluation: From *National Health Service Standards*, 2nd edn., Australian Commission on Safety and Quality in Healthcare. https://www.safetyandquality.gov.au/sites/default/files/2021-05/national_safety_and_quality_health_service_nsqhs_standards_second_edition_-_updated_may_2021.pdf

Figure 2 provides an example of the actions (or proof) health services may demonstrate to obtain accreditation in this standard.

The question remains, how do services partner with people? Beyond consumer committees and feedback sessions, researchers have found that consumer engagement in more difficult activities, such as governance, are often neglected (Farmer et al., 2018). This is particularly so in minority population group (De Freitas et al., 2015), raising questions as to the reality of partnering for system redesign. Indeed, the healthcare system is a complex one, and people from minority population groups, those who live with chronic disease and those who are vulnerable to health inequity, find access to care and health provision most difficult. This is known as structural vulnerability (Mold, 2017), a point which person-centred systems could address.

Importantly, the National Standards, consumer rights and these other forms of discourse are being produced within the vacuum of new public management. Health services, above all, are entities with business-like functions, systems which must operate within financial and political boundaries. The system is designed to be ‘well-run’ (Rudge, 2011: 167), where the processes force consumer engagement in ways that concur with pre-defined structures.

Certainly, power imbalances, specifically in consumer recruitment, meeting structures and decision-making, are a known barrier to authentic engagement (Merner et al., 2023). When scrutinised further, the national health service standards do little (if nothing) to assess consumer redesign, with measurements for this suggested in Table 2 (bolded for emphasis).

**Table 2.** Suggested measures for partnering with consumers.

Review of relevant documents regarding the **involvement of consumers** in organisational design and governance, such as terms of reference, meeting records and consultation processes
Surveys, interviews or discussions with consumers about their **experience** of being **involved** in organisational design and governance
Surveys, interviews or discussions with the workforce about the **involvement of consumers** in organisational design and governance

Source: ACSQHC (2018: 17).

Interestingly, the only time change is discussed, is in the context of workforce education where ‘the health service organisation works in partnership with consumers to incorporate their views and experiences into training and education for the workforce’ (ACSQHC, 2018: 20). This seems to circumnavigate the need to design structures with person-centred principles in mind.

We see examples of this form of dissociation between goal and target, and the rhetoric of PCC in other forms of discourse. Indeed, state-level drivers such as Clinical Excellence Division in New South Wales have included PCC as one of the six dimensions of quality care. Defined as ‘putting the patient at the centre of the care *system*’ (Clinical Excellence Division, 2020), the resources available to support PCC are located with health service *staff*, rather than within any structure or system design. The resources include person-centred communication, dementia care, health literacy and handover resources, to name a few (Clinical Excellence Division, n.d.). Certainly, facilitating practice change is a simpler, cost-effective task than re-structuring a system which relies on siloed, linear processes for care and for funding (Rycroft-Malone, 2018).

Through this analysis, it is understood that PCC is defined, at least in part, by its location of the person within the centre of the system. It includes elements of system design and governance, yet achieving this level of engagement and change is not simple, especially in the context of an increasingly complicated healthcare system. However, locating PCC in the realm of clinical practice, provides health services an opportunity to better define, demonstrate and measure such a concept.

### Person-centred *practice* – the nursing profession

On the flip side of PCC as a system design, the notion of PCP is evident. While it is not always referred to as this, the discourse alludes to personal staff attributes, and practice elements, which describe centredness as a way of practising care. There are many ways in which this is embodied.

Australia has adopted the International Council of Nurses (2021: 8, 26) Code of Conduct and in doing so, has assumed some responsibility in delivering ‘*evidence-informed, person-centred care, recognising and using the values and principles of primary health care and health promotion across the lifespan’*. Of note, the Code defines PCC as:

Valuing and respecting the characteristics, attributes and preferences of the patient, such as cultural and religious beliefs, and incorporating them into the planning and implementation of nursing care, services or programmes design.

This definition is careful to include elements of system design along with the attributes of nurses.

How nurses practise within Australia is (partly) defined by the Registered Nurse Standards for Practice (Nursing and Midwifery Board of Australia, 2016). Professional relationships are described as person-centred, in that the nurse establishing boundaries, communicates with respect, providing support and advocacy to individuals and families. These attributes are depicted as flowing through the entire care trajectory, and creating an ethos of practice which assists nurses in conducting assessments, developing plans, providing safe, responsive care and evaluating care outcomes (Nursing and Midwifery Board of Australia, 2016: 6). PCC in this context is defined as a:

. . .collaborative and respectful partnership built on mutual trust and understanding through good communication. Each person is treated as an individual with the aim of respecting people’s ownership of their health information, rights and preferences while protecting their dignity and empowering choice. Person-centred practice recognises the role of family and community with respect to cultural and religious diversity.

However, standards for practice, much like modern healthcare, have evolved. Indeed, previous research has demonstrated that the progression of Competency Standards to Standards for Practice in Australia realigned nursing practice to fit more within the fiscal ideologies of the healthcare system (Byrne, 2022). For example, the Competency Standards asked nurses to identify insufficient resource and recommend changes to policies, where care was impeded (NMBA, 2006). The Standards for Practice now asks nurses to use resources ‘effectively and efficiently’ (NMBA, 2016: 4–5). Of note here, is that the previous Competency Standard 2.4f ‘recommends changes to policy, procedures and guidelines when rights are compromised’ was removed entirely in the new iteration (Byrne, 2022). This is important for PCC and highlights that the ability to practice PCC is impacted by how it is positioned, and by the underlying ideations within the organisation.

This is further articulated by the Australian College of Nursing, the peak professional body for nursing, which suggests that PCC is a marriage of personal and professional attributes, along with the care environment in which nurses work. Attributes include those discussed above, along with professional competence and self-awareness. The environment that best supports PCC is described as including appropriate skills mix (adequately trained staff), transformational leadership, shared power and the potential for innovation among others (Australian College of Nursing, 2020). Importantly, nurses at the forefront of practice have little space to enact or influence change. Policy, reporting lines, care structures and processes do not allow nurses to influence the environment to which they belong; this is a form of structural violence that nurses must endure (Rudge, 2011).

From those enacting PCC, a theory to practice gap exists. Byrne et al. (2020) found that the gap between practising PCC and its theoretical underpinning was evident in the challenges around translating the attributes into a concrete concept. Many nurses describe the practice of such care as an ‘extra’, such as going the extra mile or doing little extras (Edvardsson et al., 2010, 2014), yet it is a required feature of nursing practice. Nurses are taught the concepts of PCC in their preparation and training and are required to demonstrate this in meeting the Standards of Practice for professional registration; however, post-graduation, the reality of nursing is not always conducive to PCC. Lack of time, managing coworker relationships, placing priority on technical capability and the complexity of policy and procedure widen the gap from theory to practice (Alharbi et al., 2023; Ko & Kim, 2022). Furthermore, nurses are constrained by workforce shortages, scope of practice, task orientated checklists, workflows including entry and exit points of care and many other influences. Nurses practise in the new public management void, and they themselves are seen as resources, where care is rationed, hours are accounted for and tasks are audited as part of hospital accreditation (ACSQHC, 2012; Moradi et al., 2023). While this is certainly a complex issue, disconnecting PCC from PCP allows us to closely examine the barriers and opportunities of improving care.

### Traversing the two – barriers and opportunities

The location of PCC itself is important, as the cultural, social and political norms permeate its meaning and understanding, as well as its operationalisation. PCC is located in a complex healthcare system, founded on the principles of new public management. Nurses too are located within this intricate healthcare system, where practice is heavily influenced and managed, and little power is afforded to make meaningful change. What is clear, is that PCC and PCP are two sides of the one coin, working hand in hand to provide care to individuals. Yet, questions remain: What do nurses need to deliver PCC? Knowing the complexity of healthcare and the barriers at play, has enough been done to change the system to support PCC?

The system is a mammoth entity. The funding structures, agendas, strategies, rules and regulations have a top-down approach on individuals. Fiscal management of services informs all decision-making, including the models of care which dictate access and equity of care. Staff within this system (particularly nurses) usually accept their role without question, understanding the system as a fixed one, where people move in, through and out. System redesign often does little to change core structures such as funding flows, access to care, care coordination and continuity of care. While more value-based healthcare models have been proposed, implementation is slow, if not stagnant (Dawda et al., 2022).

While we await such changes to an ever-ailing healthcare system, services could look to pockets of expertise in the nursing profession, the largest cohort of healthcare professions, to lead the way in system reform and practice.

Person-centred reporting frameworks currently exist, which include elements of housing, education, employment, income, health, safety and social support (AIHW, 2022b). Given that these are in line with the social determinants of health (WHO, n.d.b), healthcare systems could align with such a scope. Certainly, there are nurse-led models of care that do exactly this, for example nurse navigators in Queensland (Harvey et al., 2020), community nursing (Blay et al., 2022) and rural and remote nurse generalists (Department of Health and Aged Care, 2023). These models of care work at the very margins of the system, helping people to traverse the healthcare landscape, integrating and coordinating care, delivering PCC by definition.

Better utilising nursing expertise and experience is precisely the scope of the Australian College of Nurses white paper – *A New Horizon for Health Service: Optimising Advanced Practice Nursing* (ACN, 2019: 13, 22).

New approaches must be established to put the patient, not the provider, at the centre of care . . . Health service innovation must be pragmatic. Testing new service models is essential in advance of large-scale reform initiatives, the service model in this proposal is advanced practice nursing.

Better utilising nursing expertise could provide an opportunity to identify and address service gaps, though caution must be taken that nurses are not simply used to plug these gaps; PCP is only one side of the coin, the other is the system within which it lives. Working models exist with lived detailed experience on the challenges that vulnerable people face in accessing and navigating the healthcare landscape. Opportunities for improvement abound in the experiences of these nursing services.

Discursively, PCC as a function of the system is clear; however, the tension lies in how this is positioned and discussed in relation to practice. While it is appreciated that systems must enable PCC to occur, more could be done to highlight PCC as a system change process. Better aligning the concept of PCC to the system, and moving it away from practice, may allow for more detailed and specific measurements to be fostered by accrediting agencies, and therefore health systems. Here is where policy could make a great difference to the lives of those seeking care.

## Conclusion

This discourse analysis aimed to explore and interrogate person-centred nursing care by investigating theory development, along with the social, cultural and political epoch in which it exists. Through ordering the discourse, a tension between PCC and PCP became evident, opening a space to investigate how nurses are hindered by the system itself. Yet, the leadership of nursing in the commitment to PCC is clear. Nurses could use their experience and expertise to facilitate system improvement to better progress outcomes for those seeking care. By separating practice from system, health services are invited to pay closer attention to the larger constructs within which care exists.

**Key points for policy, practice and/or research**

• Person-centred care and person-centred practice are two separate, yet important elements of care. While focus has been placed on the attributes of nurses in practising care, changes to systems to support person-centred care are less visible.
• The tension between the system and nursing practice causes theory dilution at the point of praxis.
• Seeing person-centred care as separate to person-centred practice opens a space for care providers to look more closely at genuine engagement and system change.
• Working models of nurse-led services currently exist, providing abundant opportunities for services to leverage this expertise in identifying and rectifying known barriers to care.
